# MRI can help differentiate Ménière’s disease from other menieriform diseases

**DOI:** 10.1038/s41598-023-49066-5

**Published:** 2023-12-06

**Authors:** Jinye Li, Long Li, Xianwen Jin, Na Hu, Xiao Kong, Linsheng Wang, Xiaoqin Li, Weiqiang Dou, Lixin Sun, Chuanting Li, Ruozhen Gong

**Affiliations:** 1 Department of Radiology, Shandong Provincial ENT Hospital, Shandong University, 4 Duan Xing-Xi Road, Jinan, China; 2 Hospital office, Shandong Provincial ENT Hospital, Shandong University, 4 Duan Xing-Xi Road, Jinan, China; 3 Department of Radiology, Shandong Maternal and Child Health Care Hospital, Jinan, People’s Republic of China; 4 GE Healthcare, MR Research China, Beijing, 100000 People’s Republic of China; 5 Department of Radiology, Shandong Provincial Hospital, Shandong University, 324 Jing Wu Wei-Qi Road, Jinan, China; 6 Gong Ruozhen Innovation Studio, Shandong Provincial Hospital, Shandong University, 324 Jing Wu Wei-Qi Road, Jinan, China

**Keywords:** Medical research, Neurology

## Abstract

It is difficult to distinguish other pathologies mimicking Ménière’s disease (MD) clinically. This study aims to investigate the differences of imaging findings and features between MD and other menieriform diseases via intravenous gadolinium-enhanced magnetic resonance imaging (MRI). 426 patients with menieriform symptoms, including MD, vestibular migraine (VM), and vestibular schwannoma (VS), underwent 3D-FLAIR and 3D-T2WI MRI 6 h after the intravenous gadolinium injection. MR images were analyzed for inner ear morphology, perilymphatic enhancement (PE), EH and other abnormalities. EH was observed at a higher rate in MD patients (85.71%) than patients with other menieriform diseases (VM group = 14.75%, VS group = 37.50%). The prevalence of unilateral EH as well as both cochlear and vestibular EH showed significant differences between MD and VM groups. The prevalence of cochlear EH (I and II) and vestibular EH (II and III) was different between MD and VM groups. The prevalence of PE was higher in MD than VM group. The degrees of cochlear and vestibular hydrops were higher in the definite than probable MD group (*P* < 0.05). Using these imaging features, MRI can be used to help differentiate MD from other menieriform diseases.

## Introduction

Ménière’s disease (MD) is characterized by clinical symptoms of recurrent, fluctuating low-frequency sensorineural hearing loss, vertigo attacks, tinnitus and/or aural fullness^1^. It is, however, difficult to distinguish other pathologies mimicking MD clinically, such as vestibular migraine (VM)^2^ and vestibular schwannoma (VS)^3^, from MD. In clinical practice, patients rarely present with typical textbook cases. Additionally, there is an overlap of key symptoms between MD and other menieriform diseases^2^. Furthermore, some authors have suggested that MD may coexist with other menieriform diseases^4^. MD symptoms may be temporary and incomplete, especially in the first year of duration for disease^5^. Approximately 9% of MD cases, the interval time from the onset of vertigo to hearing loss was last for more than 10 years^6^. The diagnostic criteria for MD usually applied in clinic are based on typical symptoms (referring to the diagnostic criteria in 2015^7^). Although the clinical MD diagnosis can be supplemented with a series of audio-vestibular tests, such as audiometry, VEMP and caloric testing. However, diagnosis remains complicated due to the lack of a gold-standard diagnostic test^6^. Its pathological hallmark is the underlying endolymphatic hydrops (EH)^8^. EH is a condition characterized by distention of the structures of cochlear duct, saccule, utricle, and ampullae filled with endolymph, which tightly correlates with the symptoms of MD observed on temporal bone analysis^9,10^. However, EH may be due to various etiologies, for instance, trauma^1^, viral infection and autoimmune processes^11^, and electrolyte imbalance^12^. Moreover, according to histopathology, not all individuals with EH have symptoms of MD, nor do all individuals with MD have EH^13^. Recently, an extensive use of gadolinium contrast-enhanced MRI has enabled a depiction of EH in a living human^14^ and allowed an assessment of the degree of EH presented in the vestibule and cochlea^13,15,16^. After intravenous gadolinium chelate agent administration, delayed three-dimensional fluid-attenuated-inversion-recovery (3D-FLAIR) MRI can detect EH in patients with audio-vestibular symptoms, providing new insights into inner ear diseases. Moreover, a few studies have proved that the increased perilymphatic enhancement (PE) may be another discriminating parameter for MD^16,17^. However, to date, few studies have been reported in distinguishing MD from other menieriform diseases based on small sample. Therefore, the main goal of this study was to investigate the differences of imaging findings including the frequency, distribution, and severity of EH, visual PE, and other imaging features between MD and other menieriform diseases via MRI, and to further determine if MRI could assist clinicians for differential diagnosis of these diseases.

## Materials and methods

### Subjects

The retrospective clinical study was approved and the requirement for informed consent was waived by the institutional review board of Shandong Provincial ENT Hospital (certificate number: XYK 20,200,104). All study methods were performed in accordance with the Declaration of Helsinki and local regulations. In this study, 426 patients (852 ears; 231 women, 195 men; mean age = 51.84 ± 12.87 years) with menieriform symptoms, for instance, fluctuating hearing loss, tinnitus, vertigo, aural fullness, or a combination of these symptoms, who visited our hospital, were recruited consecutively from January 2018 to November 2019. Finally, these patients were divided into 3 groups: definite or probable MD^7^ (MD group, n = 357: definite MD, n = 310, probable MD, n = 47), definite or probable vestibular migraine patients^18^ (VM group, n = 61) (Table 1), and intracanalicular vestibular schwannoma patients (VS group, n = 8).

**Table 1:** Diagnostic criteria of Ménière’s disease and vestibular migraine.

Definite Ménière’s disease	Probable Ménière’s disease	Definite vestibular migraine	Probable vestibular migraine
Two or more spontaneous episodes of vertigo, each lasting 20 min to 12 h	Two or more episodes of vertigo or dizziness, each lasting 20 min to 24 h	At least five episodes with vestibular symptoms of moderate or severe intensity, lasting 5 min to 72 h	At least five episodes with vestibular symptoms of moderate or severe intensity, lasting 5 min to 72 h
Audiometrically documented low- to medium-frequency sensorineural hearing loss in one ear, defining the affected ear on at least one occasion before, duringor after one of the episodes of vertigo	Fluctuating aural symptoms (hearing, tinnitus or fullness) in the affected ear	Current or previous history of migraine with or without aura according to the ICHD	Only one of the criteria B and C for vestibular migraine is fulfilled (migraine history or migraine features during the episode)
Fluctuating aural symptoms (hearing, tinnitus or fullness) in the affected ear	Not better accounted for by another vestibular diagnosis	One or more migraine features with at least 50% of the vestibular episodes	Not better accounted for by another vestibular or ICHD diagnosis
Not better accounted for by another vestibular diagnosis	–	Not better accounted for by another vestibular or ICHD diagnosis	–

*ICHD* International classification of headache disorders.

Eligibility criteria included (1) age of at least 18 years (2) a history of clinical menieriform symptoms and (3) successfully completed 3D-FLAIR and 3D-T2WI examination with delayed acquisition at least 6 h after intravenous administration of gadolinium chelate agent. The exclusion criteria were also applied, including (1) head and neck trauma or neoplasm, (2) prior ear surgery of any kind, (3) serious psychiatric disease, hematological disease, prior treatment with chemotherapy agents or other immunosuppressive drugs (4) the diseases with intralabyrinthine space occupied by mass, (5) middle ear pathology that could impede local contrast uptake, (6) MR-related contraindications such as cardiac pacemakers or claustrophobia, a history of allergies to gadolinium contrast agents.

All patients underwent a double-dose (0.4 ml/kg) intravenous injection of gadolinium contrast agents. After 6 hours^19^, the corresponding MR images were acquired with 3D-FLAIR. The optimal image contrast reported previously at 4 h under single dose (0.2 ml/kg)^4^ was not observed under double-dose injection in our daily work. According to the previous work^19^, 6 h was identified an optimal scan interval after double dose contrast administration in 3D-FLAIR images and thus applied in this study. In addition, 3D-T2WI imaging was also performed to obtain reference anatomic images of the labyrinthine fluid space.

### MR experiments

All MRI scans were performed on a 3 T clinical scanner (Discovery 750w, Waukesha, USA) with a 19-channel phased-array head-neck coil. The parameters for 3D-FLAIR as follows: inversion time (TI) = 2500 ms; repetition time (TR) = 9000 ms; echo time (TE) = 130 ms; echo train length (ETL) = 140; matrix size = 256 × 256; NEX = 2; bandwidth = 36 kHz; field of view (FOV) = 21 cm × 16 cm and slice thickness = 1.6 mm, overlap = 50%. The scan time was 5 min 46 s. The parameters for 3D-T2WI as follows: TR = 2500 ms; TE = 102 ms; ETL = 120; matrix size = 256 × 256; NEX = 2; bandwidth = 42 kHz; FOV = 21 cm × 16 cm and slice thickness = 1.0 mm, overlap = 50%. The scan time was 2 min 41 s.

### Data collection and analysis

All MRI data were analysed in a vendor-provided ADW 4.6 workstation.

Two radiologists, with respective 10 and 15 years of working experience in otolaryngology, were blinded to the clinical data and independently employed for MR imaging analysis. The time interval between reading sessions for the two radiologists was more than 1 week. The following aspects were evaluated, including (1) Inner ear malformation in the vestibule, cochlea, and SCC (semicircular canal) was assessed for patients; (2) Visually increased PE means that visually higher PE compared with the contralateral ear or as matching the visual signal intensity of acute blood-labyrinth barrier breakdown/acute inflammation ear, which was a modified definition described by van Steekelelenburg et al.^17^; (3) The EH in unilateral or bilateral sides, and in cochlear, vestibular, or both cochlear and vestibular parts of the inner ear were recorded; (4) We assessed the degree of EH in the vestibule and cochlea by comparing hypointense endolymph space with hyperintense perilymph space in the axial plane separately. According to Baráth et al.^15^, cochlear EH can be classified as none, grade I, or grade II. For the degrees of vestibular EH, a modified four-stage grading system described by Bernaerts et al.^16^ was applied^20^. Moreover, cochlear and vestibular EH (I), vestibular EH (II), and cochlear EH (II) and vestibular EH (III) were separately defined as mild, moderate, and significant hydrops; (5) The lesions of other locations were also visually assessed, including the internal auditory canal, cerebellopontine angle and brain. For not agreed evaluation between both radiologists, a consensus judgment was reached after discussion.

Weighted Kappa (κ) statistics were applied to assess the interobserver agreement of evaluating the EH and other abnormal imaging characteristics within the inner ears over two radiologists. Results less than 0.20 were interpreted as poor; 0.21 to 0.40 were fair; 0.41 to 0.40 were moderate; 0.61 to 0.80 were good; or 0.81 to 1.00 excellent. Additionally, the Chi-square test and Fisher's exact test were used to analyze the data in EH, PE and other abnormal imaging characteristics between definite and probable MD patients, and between MD and VM groups. The above statistical analyses were performed using IBM SPSS 20.0 software (IBM, Chicago, IL, United State). A *P* < 0.05 was considered statistically significant.

## Results

### Endolymphatic hydrops

EH was present in all MD, VM and VS groups. The percentages of patients were compared among MD, VM and VS groups in terms of EH occurred in unilateral side, bilateral sides, cochlear part, vestibular part and both cochlear and vestibular parts of the inner ears, respectively (Table 2). Finally, these were 306 MD patients (346 ears) with EH (definite MD, n = 276 [313 affected ears], probable MD, n = 30 [33 ears]), 9 VM patients (11 ears) with EH, and 3 VS patients (4 ears). Meanwhile, they were also compared among these three groups at different degrees of EH in the labyrinthine (Table 3).

**Table 2:** Analysis for detection of endolymphatic hydrops in patients with Ménière’s Disease or other menieriform diseases.

	Ménière’s disease (n = 357)	Vestibular migraine (n = 61)	Chi-square test/Fisher's exact	*P* value*	Vestibular schwannoma (n = 8)
EHu	266 (74.50%)	7 (11.48%)	91.369	< 0.001	2 (25.00%)
EHb	40 (11.20%)	2 (3.28%)	2.797	0.094	1 (12.50%)
EHc	8 (2.24%)	0 (0.00%)	–	0.610	1 (12.50%)
EHv	34 (9.52%)	8 (13.11%)	0.743	0.389	0 (0.00%)
EHc + v	264 (73.95%)	1 (1.64%)	114.296	< 0.001	2 (25.00%)

*EHu* unilateral EH, *EHb* bilateral EH, *EHc* cochlear EH, *EHv* vestibular EH, *EHc* + *v* cochlear and vestibular EH.

*P values were obtained by separately comparing the differences in endolymphatic hydrops between patients with Ménière’s disease and with vestibular migraine. Due to limited sample size in vestibular schwannoma, no such statistical analysis was performed. Values are presented as No. of patients.

**Table 3:** Analysis for the degree of endolymphatic hydrops in patients with Ménière’s Disease or other menieriform diseases.

	Ménière’s disease (n = 714)	Vestibular migraine (n = 122)	Chi-square test/Fisher's exact	*P* value*	Vestibular schwannoma (n = 16)
None cochlear EH	420 (58.82%)	121 (99.18%)	74.315	< 0.001	12 (75.00%)
Cochlear EH (I)	174 (24.37%)	1 (0.82%)	34.915	< 0.001	1 (6.25%)
Cochlear EH (II)	120 (16.81%)	0 (0.00%)	–	< 0.001	3 (18.75%)
None vestibular EH	382 (53.50%)	112 (91.80%)	63.234	< 0.001	13 (81.25%)
Vestibular EH (I)	71 (9.94%)	8 (6.56%)	1.397	0.237	0 (0.00%)
Vestibular EH (II)	140 (19.61%)	2 (1.64%)	23.858	< 0.001	1 (6.25%)
Vestibular EH (III)	121 (16.95%)	0 (0.00%)	–	< 0.001	2 (12.50%)

*P values were obtained by separately comparing the differences in endolymphatic hydrops between patients with Ménière’s disease and with vestibular migraine. Due to limited sample size in vestibular schwannoma, no such statistical analysis was performed.

Values are presented as No. of ears.

### Detection of Endolymphatic Hydrops

In MD group, the rate of EH (85.71%) was higher than that in the other two groups (VM group = 14.75%, VS group = 37.50%). Moreover, the prevalence of unilateral EH was higher than that of bilateral EH in each of three groups, especially in MD group. The prevalence of unilateral EH showed a significant difference between MD and VM groups (*P* < 0.001). Also, the prevalence of unilateral EH in MD group was higher than that in VS group (Table 2).

Furthermore, the prevalence of both cochlear and vestibular EH was higher than that of single cochlear EH or single vestibular EH only in MD and VS groups, and the prevalence of single vestibular EH (9.52%) was higher than that of single cochlear EH (2.24%) in MD group. However, the difference was that no patient showed single cochlear EH in VS group. In VM group, the prevalence of single vestibular EH (13.11%) was higher than that of both cochlear and vestibular EH (1.64%) and no patient showed single cochlear EH. The prevalence of both cochlear and vestibular EH showed a significant difference between MD and VM groups (*P* < 0.001). Additionally, the prevalence of both cochlear and vestibular EH in MD group was higher than that in VS group (Table 2). EH occurred in uni- and bi-lateral sides and different parts of the inner ear showed no significant difference between probable and definite MD groups (*P* > 0.05).

### Degree of endolymphatic hydrops

**Figure 1:**
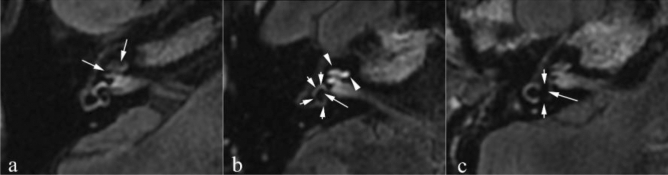
Cochlear and vestibular endolymphatic hydrops with different degrees were shown. High-resolution 3D-FLAIR images were obtained 6 h after intravenous gadolinium injection for patients with Ménière’s disease. A 45-year-old man with right Ménière’s disease (**a**): Axial 3D-FLAIR image showed that the scala vestibuli was fully obliterated due to the dilated cochlear duct (arrow) (cochlear hydrops grade II). A 49-year-old man with right Ménière’s disease (**b**): Axial 3D-FLAIR image showed a confluence of the saccule and utricle (long arrow) with a continuous peripheral rim enhancement of the perilymphatic space (short arrows) (vestibular hydrops grade II). Note: abnormal cochlear perilymphatic enhancement (arrowhead) was shown on the same side. A 48-year-old woman with right Ménière’s disease (**c**): Axial 3D-FLAIR image showed that the perilymphatic enhancement was no longer continuous. There was a full obliteration of the bony vestibule, and the perilymphatic enhancement was no longer visible (short arrow) (vestibular hydrops grade III).

MD group presented EH with varied degrees. In VM group, the degrees of EH were predominantly mild or moderate, including cochlear EH (I) and vestibular EH (I and II), with no significant EH (cochlear EH [II] or vestibular EH [III]) observed (Fig. 1 and Table 3). The prevalence of cochlear EH (I and II) and vestibular EH (II and III) showed significant differences between MD and VM groups (*P* < 0.001; Fig. 1 and Table 3). Moreover, the present study demonstrated that the degrees of cochlear and vestibular EH in definite MD were higher than those in probable MD (*P* = 0.006; Table 4).

**Table 4:** Analysis of cochlear and vestibular endolymphatic hydrops in patients with probable and definite Ménière’s disease.

	Cochlea				Vestibule				
	None	Grade I	Grade II	*P* value	None	Grade I	Grade II	Grade III	*P* value
Probable (n = 94)	67 (71.28%)	17 (18.09%)	10 (10.64%)		64 (68.09%)	11 (11.70%)	10 (10.64%)	9 (9.57%)	
Definite (n = 620)	353 (56.94%)	157 (25.32%)	110 (17.74%)	0.029	318 (51.29%)	60 (9.68%)	130 (20.97%)	112 (18.06%)	0.006

Values are presented as No. of ears.

### Perilymphatic enhancement

**Figure 2:**
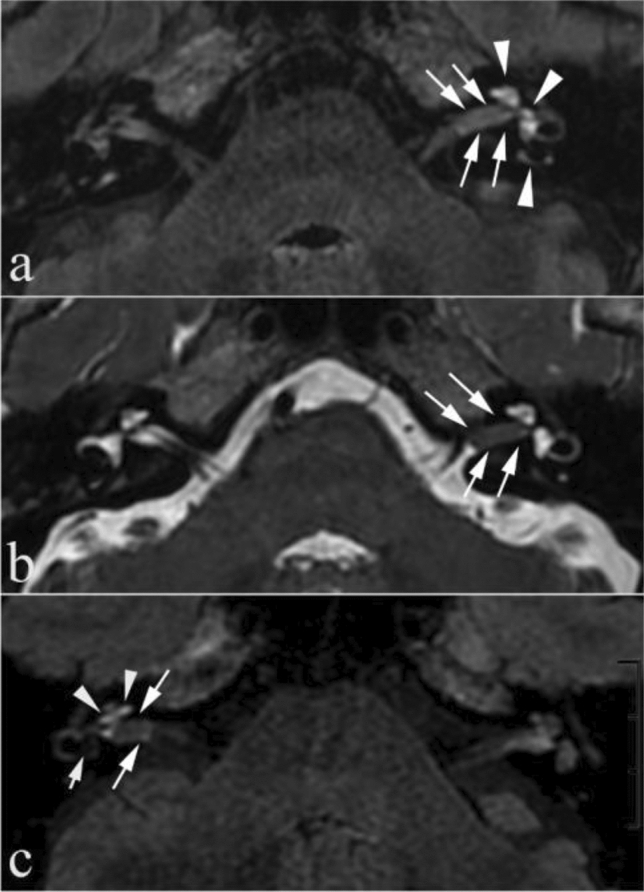
A 50-year-old woman with left vestibular schwannoma (**a**, **b**) and a 49-year-old man with right vestibular schwannoma (**c**) in the internal auditory canal. The inner auditory canal schwannoma presented relatively equal hyperintense (long arrows) on intravenous gadolinium-enhanced 3D-FLAIR (**a**, **c**) and asymmetric hypointense (long arrows) on 3D-T2WI (**b**) compared with the contralateral side, and the nerves in the internal auditory canal were unclear. Axial 3D-FLAIR image (**c**) showed vestibular hydrops grade III (short arrow). Note: abnormal cochlear (arrowhead) and/or vestibular (arrowhead) perilymphatic enhancement was shown on the same side compared with the contralateral side (**a**, **c**).

In MD group, the rate of visual PE (77.87%) was higher than that in the other two groups (VM group = 22.95%, VS group = 75.00%) (Table 5). Visual PE in affected side inner ear was shown in Figs. 1b, 2a,c.

**Table 5:** Analysis of PE and other abnormal imaging characteristics in patients with Ménière’s disease or other menieriform diseases.

	Ménière’s disease (n = 357)	Vestibular migraine (n = 61)		Chi-square test/Fisher's exact	*P* value*	Vestibular schwannoma (n = 8)
PE	278 (77.87%)	14 (22.95%)		74.625	< 0.001	6 (75.00%)
IEM	3 (0.84%)	0 (0.00%)		–	1.000	0 (0.00%)
ME	4 (1.12%)	3 (4.92%)		2.548	0.110	0 (0.00%)
SCCLSL	4 (1.12%)	0 (0.00%)		–	1.000	0 (0.00%)

*PE* perilymphatic enhancement; *IEM* inner ear malformation; *ME* middle ear effusion without clinical features of active inflammation; *SCCLSL* semicircular canal local signal loss.

*P values were obtained by separately comparing the differences in PE, IEM, ME and SCCLSL between patients with Ménière’s disease and with vestibular migraine. Due to limited sample size in vestibular schwannoma group, no such statistical analysis was performed.

Values are presented as No. of patients.

### Other abnormal imaging characteristics

**Figure 3:**
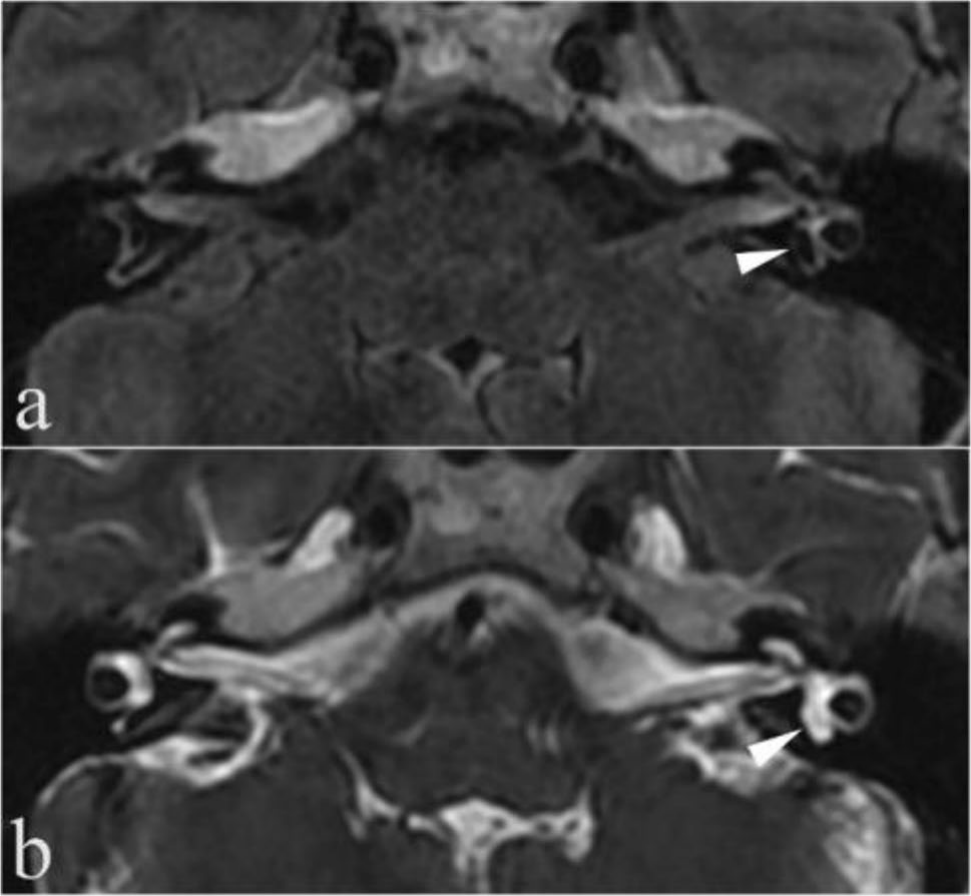
A 62-year-old woman with left Ménière’s disease and posterior semicircular canal dysplasia (**a**, **b**). 3D-FLAIR (**a**) and 3D-T2WI (**b**) showed left posterior semicircular canal dysplasia (arrowheads). The endolymph compartment occupies more than 50% of the short and enlarged posterior semicircular canal.

Other abnormal imaging characteristics, including inner ear malformations (e.g., vestibular and semicircular canal malformations) (Fig. 3), middle ear effusion without clinical features of active inflammation (middle ear fluid with hyperintense signal on T2WI) and the lateral part of the lateral SCC or all SCC signal loss sometimes, co-occurred in the same patients (Table 5). The “other abnormal imaging characteristics” were exceptionally heterogeneous. Therefore, the results may be potentially biased. The comparisons of these characteristics among MD and other menieriform diseases were illustrated in Table 5.

All other abnormal imaging characteristics appeared a low prevalence in MD group. Inner ear malformations and SCC local signal loss were not present in VM and VS patients. Also, for VS patients, middle ear effusion without clinical features of active inflammation was not present. In addition, other abnormal imaging characteristics, including inner ear malformations, middle ear effusion without clinical features of active inflammation, and SCC local signal loss, showed no significant difference between MD and VM groups (both* P* > 0.05). The sample size of VS group was relatively small and the corresponding results may thus be potentially biased. However, this kind of mass was equal hyperintense on intravenous gadolinium-enhanced 3D-FLAIR and asymmetric hypointense on 3D-T2WI relative to the contralateral side (Fig. 2).

## Discussion

In this study, we systematically investigated the differences of imaging findings and features, including EH, visual PE, inner ear malformations, middle ear effusion without clinical features of active inflammation, and SCC local signal loss, for patients with MD and other menieriform diseases by means of 3D-FLAIR and 3D-T2WI performed 6 h after intravenous injection of gadolinium contrast agents. With obtained significant imaging differences, MRI can be considered to assist clinicians for differential diagnosis between MD and other menieriform diseases.

### Endolymphatic hydrops

EH was observed more often in MD patients. In MD group, the rate of EH was much higher than that in the other two groups. Temporal bone histopathology studies show that EH, a ballooning of the endolymph, is the primary pathophysiologic finding in MD^21^. By temporal bone autopsy, EH was detected in every patient with a history of definite MD. Additionally, Nakashima^22^ and Fiorino et al.^23^ demonstrated that EH via MRI was present in every patient with definite MD, being higher than the finding reported in our study (89.03%). Foster CA et al.^24^ inferred that EH might cause MD, but EH alone is insufficient to cause MD. This indicates that asymptomatic EH can become symptomatic MD due to one or more additional cofactors, and vascular risk factors should be studied as possible cofactors. Therefore, if EH together with additional cofactors causes MD, the rate of EH in MD is then higher than that in other groups.

Additionally, the prevalence of unilateral EH was higher than that of bilateral EH in all groups, especially in MD group. This finding is similar to most previous studies^25,26^. In this present study, the prevalence of unilateral and bilateral EH in definite MD group were both higher than that of probable MD group. It has been well acknowledged that unilateral MD is highly likely to progress to bilateral MD over time^27^. Furthermore, our results revealed that for MD patients, EH could occur at the cochlea, vestibule, or both regions together, while single cochlear EH was not found in VM patients and single vestibular EH was not found in VS patients. According to the literature studies and present work, the prevalence and occurred regions of EH in VM patients were different^25,28–30^. Because the sample sizes in these studies were relatively small, the results may be biased. Moreover, in our study, single-region EH (vestibular or cochlear) is a relatively rare finding although single vestibular EH is more pronounced than single cochlear EH in MD and VM groups. This is in line with a previously published study^31^, and recent studies suggest that relative to cochlear EH, vestibular EH is more specific for MD patients^26,32^. This indicate that vestibular EH can contribute to the MD symptoms, such as fluctuating hearing loss, whereas cochlear EH may form in early MD stage but rarely induces its symptoms^31^. In addition, the prevalence of both cochlear and vestibular EH was high in MD patients and showed a significant difference between MD and VM groups. Hallpike and Cairns reported a 28-year-old autopsied case^7^ and a 29-year-old autopsied case^33^ in which advanced EH appeared in early cases of MD and may indicate that EH developed rapidly from the cochlea to the vestibule.

This result revealed that every MD patient had EH with different degrees, whereas VM patients did not have significant EH (cochlear EH [II] or vestibular EH [III]). This coincided with a previously published study^25^. VM and MD patients have similar clinical manifestations and both diseases behave similarly on most vestibular tests^34–37^. Therefore, the vestibular dysfunction and transduction pathway in VM and MD may be similar, however, MD maybe more severe than VM. Although the pathophysiology of MD and VM is not fully understood, but Sun et al.^25^ reckoned that MD may be mainly due to dilation of membranous labyrinth space, while VM is mainly caused by abnormal vestibular central pathways. However, different from MD and VM groups, the prevalence of significant EH (cochlear EH [II]: 18.75%, vestibular EH [III]: 15.38%) in VS group was higher than that of mild or moderate EH (cochlear EH [I]: 6.25%, vestibular EH [I]: 0.00%, vestibular EH [II]: 7.69%), which was in accordance with a previous report^30^.

The present study showed that compared with probable MD group, cochlear and vestibular EH in definite MD group were more severe, which was consistent with Katio et al.^38^ and Xie et al.^39^. Both studies concluded that significant degree of EH were more likely to occur in patients with definite MD than those with probable MD. Li et al.^26^ suggested that during the asymptomatic stage, mild EH is likely to be observed in both the cochlea and the vestibule. As the disease progression, the degree of EH gradually increases, especially in the vestibular EH, and the clinical MD symptoms become apparent. The presence of EH in the vestibule and significant EH in the cochlea correlated with the presence of symptomatic MD^32^. Also, as shown previously^40^, the affected ears had significantly cochlear or vestibular EH with an intense contrast-effect, which indicates that the blood-labyrinth barrier is impaired in the affected ears of MD patients.

### Perilymphatic enhancement

In addition to EH, PE was also observed more often in MD patients than in other two groups. This was same as the finding of van Steekelelenburg et al.^17^. PE was a good indicator for contrast permeability of inner ear, which can give hints about its’ blood-labyrinth barrier damage^41^. Moreover, the blood-labyrinth barrier may play a role in the process of EH in MD^42^. This demonstrated that PE may be another discriminating parameter for MD^16,17^. The PE indicated by the delayed 3D-FLAIR imaging was most likely due to three possible causes: (1) impairment of the blood-perilymph barrier^43^, (2) blockage of neuroaxonal transport mechanisms^44^ and (3) cellular immune reaction^45^. For MD and VM patients, the first cause would be the primary one for PE, or MD patients showed more severe degree of impaired blood-perilymph barrier than VS and VM groups. In comparison, all the above mentioned three causes could contribute to VS patients.

### Other abnormal imaging characteristics

Our study showed that patients with menieriform symptoms also presented inner ear malformations, middle ear effusion without clinical features of active inflammation and SCC local signal loss upon MRI. Although the small VS group might bring potentially biased results, there are indeed certain useful imaging characteristics shown to distinguish MD from VS. MD and VS are usually not challenging to be distinguished on MRI owing to the presence of a mass in VS cases. This kind of mass was equal hyperintense on intravenous gadolinium-enhanced 3D-FLAIR and asymmetric hypointense on 3D-T2WI relative to the contralateral side, which did not present in MD patients. Additionally, thin-section gadolinium-enhanced T1-weighted MRI and diffusion weighted imaging were employed to distinguish VS from other tumors or tumor-like lesions. Other abnormal imaging characteristics, including inner ear malformations, middle ear effusion without clinical features of active inflammation, and SCC local signal loss, may be incidental findings in the present study. To our knowledge, we did not searched other previous paper about the relationship of these abnormal imaging characteristics with MD. Alternatively, the difference of these abnormal imaging characteristics between the two groups was not statistically significant may due to the relatively small number of patients included in the VM group. The immune response is thought to play a central role in the pathophysiology of the MD^46,47^. The cytokines and inflammation may lead to middle ear effusion and/or labyrinthitis, the latter ossification stage can be manifested as semicircular canal local signal loss. However, there are many causes of otitis media, which may be concurrent with MD or VM. In addtion, inner ear malformations maybe associated with MD, because structural changes (e.g., vestibular aqueduct, endolymphatic duct and sac) have been implicated in the development of MD^48–50^.

One limitation of the present study was that the MR signal intensity was visually assessed by comparing with the contralateral side, which did not provide quantitative evaluation for the difference of image enhancement. However, to ensure the reliability of the corresponding signal measurement in the inner ear, every patient was evaluated twice by two experienced radiologists independently. Moreover, as the VS group was small and the “other abnormal imaging characteristics” was very heterogeneous, the corresponding results may be potentially biased. Our results should thus, be further verified in a large clinical cohort in future study.

## Conclusion

In conclusion, MRI revealed a higher prevalence of unilateral EH in patients with MD than in patients with other menieriform diseases, a more severe degree of EH and PE in patients with MD than that in patients with VM, and a lower prevalence PE in patients with VS than that in patients with MD. Using these characteristic imaging features, MRI can be used to help differential diagnosis between MD and other menieriform diseases.

## Data Availability

The datasets analyzed during the current study are available from the corresponding author upon reasonable request.
